# Variation in Root Biomass and Distribution Based on the Topography, Soil Properties, and Tree Influence Index: The Case of Mt. Duryun in Republic of Korea

**DOI:** 10.3390/plants13101340

**Published:** 2024-05-13

**Authors:** Julia Inacio Carvalho, Mark Bryan Carayugan, Lan Thi Ngoc Tran, Jonathan O. Hernandez, Woo Bin Youn, Ji Young An, Byung Bae Park

**Affiliations:** 1 Department of Forest Resources, College of Agriculture and Life Science, Chungnam National University, Daejeon 34134, Republic of Korea; juliaic@o.cnu.ac.kr (J.I.C.); macarayugan@up.edu.ph (M.B.C.); dbsdnqls95@cnu.ac.kr (W.B.Y.); 2 Forest Environment and Geospatial Technology Research Institute, Sejong 30098, Republic of Korea; tranlan1218@gmail.com; 3 Department of Forest Biological Sciences, College of Forestry and Natural Resources, University of Philippines, Laguna 4031, Philippines; johernandez2@up.edu.ph; 4 Division of Environmental and Forest Science, College of Agriculture and Life Sciences, Gyeongsang National University, Jinju 52725, Republic of Korea; ajy2656@gnu.ac.kr

**Keywords:** root distribution, sloping mountain, topography, tree density, tree influence index

## Abstract

Root biomass and distribution are influenced by abiotic factors, such as topography and soil physicochemical properties, determining belowground productivity. Hence, we investigated the variation in root biomass and vertical root distribution based on the topography, soil physicochemical properties, and tree influence index, and their relationships, across soil depths (0–10 cm, 10–20 cm, and 20–30 cm) and topographical gradients in a warm-temperate forest in Mt. Duryun, Republic of Korea. Two contrasting research sites were established: a lower slope oriented at ≤3° and an upper slope with a slope of 30°. Each site comprised eleven 400 m^2^ sampling plots from which root samples from various diameter classes (<2 mm, 2–5 mm, 5–10 mm, and >10 mm) were collected. While the bulk density increased with soil depth in the lower slope, the organic matter, available phosphorus, Ca^2+^, and Mg^2+^ showed a reversed pattern. Linear mixed-effects models generally revealed significant negative correlations between root biomass and soil pH, total nitrogen, and cation exchange capacity, particularly in small roots (βstd = −1.03 to −1.51) and coarse roots (βstd = −6.30). Root biomass exhibited a 10–15% increase in the upper slope compared to the lower slope, particularly in fine (median = 52.0 g m^2^–65.64 g m^2^) and medium roots (median = 56.04 g m^2^–69.52 g m^2^) at a 0–20 cm soil depth. While no significant correlation between root biomass and the tree influence index was found on the lower slope, a different pattern was found on the upper slope. Our results indicate that the variation in root biomass and distribution can also be explained by the differences in the soil environment and topographical positions.

## 1. Introduction

Roots regulate plant and ecosystem functions, provide resources to the soil microbial community, and influence water, carbon, and nutrient cycling [1,2]. Roots constitute a significant portion of the total biomass in forest ecosystems [3]. Specifically, fine roots assume an essential role in terrestrial carbon dynamics, supporting 38.1 Pg of the global carbon (C) in their biomass and supplying up to 60% of C to the mineral soil [4,5]. Root growth and distribution, either horizontal or vertical (e.g., along a gradient of soil depth), is controlled by the different abiotic factors (e.g., soil texture and compaction, presence of stones, slope angle, etc.) [6,7,8]. These complexities add to the challenge of accurately estimating root characteristics. Thus, there is a need for a reliable biomass assessment of tree roots to help us estimate ecosystem productivity, scale up primary production measurements, predict ecosystem functioning and tree root adaptability mechanisms, and inform management strategies in changing environments. However, the estimation of root biomass remains a challenge due to the complexities of root architecture, distribution, and turnover rates within ecosystems, especially in areas where root excavation is difficult, such as on upper slopes. Further, root biomass variation along environmental gradients is less well understood than aboveground parts, which have received substantial research [9]. 

Topography plays an important role in shaping root growth and distribution by controlling the spatial variation of soil’s physical properties and nutrients, sediment transport, drainage patterns, mass movement, and erosion rates [10,11]. Plants experience mechanical stress as a result of the combined influences of inclination, stem, and soil slope positions, to which all plants respond by altering root growth and distribution to avoid uprooting [12,13]. On steep terrain, plant roots tend to elongate uphill, and as the angle increases, so does root growth because of gravitational force [14]. Mechanical forces active on steep slopes influence root system development by developing a specific root asymmetrical architecture as a result of preferential lateral root emergence and elongation in the upslope and downslope orientations [12]. Moreover, lower slopes generally have a more fertile soil environment than upper slopes [15,16,17,18] because nutrients are transported downhill from the upper slope, resulting in an eventual accumulation downslope [19,20]. Other geohydrologic processes may also transport nutrient-deficient soil mass from the upper to lower slopes, reducing the availability of topsoil nutrients [21]. Thus, studies on the potential relationships among topography, soil physicochemical properties, and plant root response will enhance our understanding of root dynamics along topographical and soil resource gradients. 

The variation in fine root biomass can also be explained by the immediate soil environment, highlighting the complex interaction between soil physicochemical properties and root development. When facing low-nutrient conditions, plants invest more in increasing fine root production to improve nutrient foraging efficiency [22,23]. Generally, fine root biomass decreases as soil fertility increases, and the relationship may vary by species and site conditions [24,25]. Moreover, soils contain abundant rock fragments of various sizes, which significantly impact soil properties and root growth [26]. The spatial distribution, size, and quantity of the rock fragments in the soil can regulate water retention, infiltration, and gas exchange processes, and also influence nutrient dynamics and erosion susceptibility [27,28], all of which are influenced by topography [29,30,31]. Fine root biomass may be limited on stony slopes as a consequence of restricted rooting space [32,33] and a reduced proportion of fine-earth particles that retain both soil moisture [29,34,35] and nutrients [26,36]. Because different ecosystems with varying species compositions and structures may respond differently to environmental gradients, investigating the relationship between soil rock fragments, soil physicochemical properties, and root biomass can help us describe root dynamics and functioning in a changing landscape. 

Root growth and distribution are also influenced by the size and proximity of nearby trees primarily through competition for resources, as coexisting plants rely on a limited supply of essential resources, such as water, nutrients, and space [37]. Hence, any major changes in the basal area [38,39], stand density [40,41,42], and other structural traits [40,43,44] of nearby trees further modify root dynamics. The proximity of trees favors competitive downward displacement of roots due to soil moisture and nutrient gradients and canopy shading [45]. Roots accumulate at select horizontal distances, depending on their size and topographic position. Moreover, existing plants can also interact with each other in positive ways (e.g., facilitation) in a particular environment. Consequently, an understanding of the effects of the size and proximity of nearby trees offers insights into competitive and facilitative interactions in belowground systems [46]. 

Consequently, the present study (a) analyzed the variations in soil physicochemical properties and rock volume between two contrasting forest stands; and (b) investigated how the variation in root biomass and vertical root distribution are influenced by the topography, soil properties, tree influence index, and their relationships at the upper and lower slopes of warm-temperate forests in Mt. Duryun, Republic of Korea. In this study, we expect to observe variations in physicochemical properties and root biomass distribution between the two slope formations due to differences in soil profiles, microenvironments, and resource gradients. Exploring the distribution of root biomass across different topographic formations and analyzing its relationship with soil chemical properties, rock content, and stand structure improves our understanding of the complexities of belowground dynamics.

## 2. Results

### 2.1. Comparative Analysis of Soil Physicochemical Properties between Lower and Upper Slope Positions

The soil texture in both research sites is predominantly silty loam (Table 1). The bulk density (BD) increased with soil depth in the lower slope, while a similar pattern was observed in the sand proportion at the upper slope. The organic matter (OM), available phosphorus (AP), Ca^2+^, and Mg^2+^ at the lower slope showed a reversed pattern. Both lower and upper slopes showed decreasing total nitrogen (TN), cation exchange capacity (CEC), and K^+^ with soil depth. 

### 2.2. Relationship between Soil Chemical Properties and Root Biomass

Linear mixed-effects models generally revealed significant positive relationships between root biomass and soil physical properties across diameter classes, except for BD, which exhibited a strong negative correlation (βstd = −0.47) with biomass of coarse roots (>10 mm) (Table 2). 

Soil pH, TN, and CEC exhibited negative correlations with root biomass, with particularly strong associations observed for fine roots (0–2 mm) (βstd = −1.03), medium roots (5–10 mm) (βstd = −6.30), and small roots (βstd = −1.51), respectively. Contrarily, K^+^ was strongly and positively correlated with medium-to-coarse root biomass. AP exhibited significant positive and negative correlation with fine and small root biomass, respectively. 

### 2.3. Root Biomass Distribution

**Figure 1:**
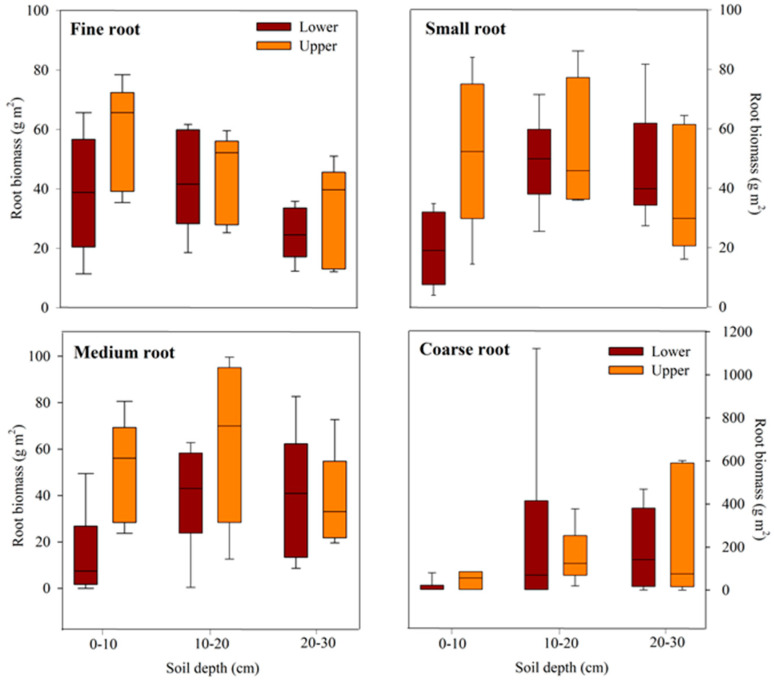
Vertical biomass distribution of fine (0–2 mm), small (2–5 mm), medium (5–10 mm), and coarse roots (>10 mm) on the lower and upper slopes of warm-temperate forests of Mt. Duryun in Haenam Province, Republic of Korea. The box represents the interquartile range, whiskers show the maximum and minimum values, and the midline represents the median.

The vertical root distribution varied significantly across soil depths (Figure 1; Table 3). Root biomass was 10–15% higher in the upper slope compared to the lower slope, particularly evident in fine (median = 52.0 g m^2^–65.64 g m^2^) and medium roots (median = 56.04 g m^2^–69.52 g m^2^) at soil depths of 0–20 cm. On the upper slope, the distribution of fine and small roots tended to decrease with soil depth, whereas on the lower slope, the distribution of medium roots tended to increase with depth. The vertical distribution of coarse roots generally remains consistent across soil depths, irrespective of the topographical position.

### 2.4. Rock Volume and Its Relationship with Root Biomass 

Rock fragments occupied 16–40% of the total ground volume on the lower slope and 15–44% on the upper slope (Table S1) with a significantly higher rock volume on the lower slope up to a depth of 10 cm (*p* = 0.04). In the lower slope, at a soil depth of 0–10 cm, there was a decrease in both medium and coarse root biomass with an increase in rock volume but this relationship is not statistically significant (Table 4). A different trend was observed in the upper slope, where fine root biomass exhibited a strong negative correlation (rho = −0.70 to −0.90) with rock distribution across soil depths. Notably, a similarly strong negative correlation (−0.84) was observed between small and medium root biomass and rock distribution, particularly those found at depths of 10–20 cm. While there may be apparent trends in the data, these strong relationships between variables observed in the upper slopes were also not statistically significant. 

### 2.5. Relationship between Tree Influence Index and Root Biomass

The results of the Spearman rank correlation analysis demonstrated that there was no significant relationship between root biomass and the tree influence index at various proximities and root diameter classes in the lower slope (Table 5). In contrast, the upper slope revealed a statistically significant relationship between these two factors. The majority of the distances on the upper slope had strong and significant relationships with fine root biomass, and the proximity of 4–5 m demonstrated the strongest negative correlation (rho = −0.78).

## 3. Discussion

### 3.1. Topography-Driven Changes in Soil Physicochemical Properties Largely Explain Root Biomass Distribution

Roots are sensitive to changes in soil physicochemical properties that may differ depending on topography [47]. In this study, the significant variation in root biomass between the upper and lower slopes, with greater root biomass in the upper slope, can be attributed to topography-driven changes in soil physicochemical properties. Specifically, we found a lower AP in the upslope than in the downslope, which could affect especially the distribution of fine root biomass. Plants produce more fine roots and generate more lateral roots in response to low P conditions to improve P absorption [48]. This also holds true for other essential resources, such as water and nitrogen [49,50]. Hence, a greater accumulation of fine roots may indicate limited essential resources in the upper slope, leading plants to direct root growth at depths of 10–20 cm for better resource exploration and uptake [51]. Greater root biomass concentration on the upper slope was also observed for pine plantations and cool-temperate forests in Japan [17,52], semi-arid forests and karst ecosystems in China [15,23], and a dry tropical forest in Thailand [53].

A decreasing distribution pattern of fine and small roots with soil depth on the upper slope may be caused by an increase in the sand proportion with soil depth. A high sand content may have resulted in a much drier and more nutrient-deficient soil as depth increased and water drained more quickly, which is not conducive to fine and small-sized roots [54,55]. This supports our study’s findings of decreasing TN, CEC, and K^+^ with soil depth in the upper slope. The spatial nutrient heterogeneity across slopes can be attributed to the transport-accumulation mechanism occurring at the research site. Nutrients are transported downward and deposited on the lower slope over time by erosion, overland flow, and other geohydrological processes, thus decreasing nutrient and water availability on the upper slope [19,20]. Contrary to our findings, soil layers deeper than 30 cm accounted for a larger fraction of fine-root standing biomass than shallower depths [56]. A previous study also revealed a drought-induced increase in fine root biomass across experimental periods [57]. This contrasting pattern observed in earlier studies could be attributed to the absence of slope effects, which can have a substantial impact on root biomass distribution patterns and responses to environmental conditions. 

The negative correlation between root biomass and rock volume, although not statistically significant, also suggests the potential influence of a high sand proportion on reducing fine and small root distribution at the deeper soil layer in the upper slope. Such a correlation may have potentially influenced root distribution more prominently as depth increases through modification in soil structure and water and nutrient retention [58]. Although the upper slope had a substantially lower rock volume than the lower slope, the presence of rocks, even in small quantities, can impede root penetration and consequently root distribution. This supports the strong negative correlation between bulk density and coarse roots, suggesting that the higher rock fragments in the lower slope may have reduced bulk density, influencing root growth. Small rock fragments can be carried by run-off, accumulating on lower slopes and leading to high rock fragment deposits on the soil surface [59,60]. Our findings are comparable with those of Zhongjie et al. [29], who reported lower rock fragments on the upper slopes than on the middle and lower slopes in the Liupan Mountains of China. Moreover, rock fragments can be favorable for plant growth within a limited range, the effects of which can be reversed beyond a site-specific threshold [34,61,62,63]. The over-deposition of rocks could weaken soil water capacity and hydraulic conductivity, which reduces available moisture for plant consumption [35]. Under these conditions, some species can promote vertical elongation, allowing them to extract a greater amount of water in an attempt to overcome resource limitations [34,62]. This leads to root biomass depletion in areas with higher rock volumes, as exemplified by the small and medium roots in our study. 

### 3.2. Competitive and Facilitative Effects Can Elucidate the Relationship between Root Biomass and Tree Influence Index

Roots interact with both abiotic and biotic factors, such as the roots of other plants. This interaction is influenced by resource gradients and by mechanisms that inhibit access of other roots to resources [64]. This can elucidate the observed statistically significant negative relationship between the tree influence index and root biomass on the upper slopes, particularly for fine roots at a 4–5 m tree distance. Such a negative relationship suggests competitive interactions between trees and fine roots for resources in the upper slope. Thus, the data also show that the tree influence at a 4–5 m tree distance decreased on the upper slope, resulting in a much greater fine root biomass. Generally, fine roots extend over certain distances away from the parent tree [44] to efficiently forage resources. Our results partially coincide with Yanai et al. [46] and Jiang et al. [25], who reported positive fine root biomass responses to trees located at a 2–5 m radius. Similarly, Jochheim et al. [65] reported fine root density to peak at a 2.5 m distance, followed by a decrease towards the tree stem and canopy gap. In stands as dense as in the upper slope, the root growth can be limited by intense belowground competition and overlapping systems, unlike in low-density stands where roots can spread longer and more uniformly [42,66]. 

Moreover, it is plausible that resource competition may have been greater uphill than downhill, explaining the lack of a significant relationship between the tree influence index and root biomass in the lower slope, which is typically more resource-rich. The observed increased proportion of sand with soil depth in the upper slope may also explain the negative relationship between the tree influence index and root biomass, intensifying resource competition due to reduced water retention and nutrient availability. In a previous study, fine root mass and length density were more than three times greater on nutrient-poor sandy soils than on nutrient-rich clay soils [67]. Several studies also reported an increase in fine root production by 21–35% under water-limited conditions [68,69]. 

The morphological differences in root diameters may also contribute to the observed result. Here, we found a significant positive relationship between the tree influence index and biomass evident only for small roots in the upper slopes. This implies that small root biomass can increase in response to an increased influence from surrounding trees, possibly due to a facilitation mechanism by the tree presence [70,71]. The presence of nearby trees within a 3–4 m distance from the pit may have ameliorated abiotic stresses caused by the limited supply of soil nutrients and moisture and harsh microclimatic conditions in the upper slope [71]. In this study, we identified *Camellia japonica* L. and *Cinnamomum japonicum* Siebold as two of the dominant tree species within a 3–4 m radius of the pit on the upper slope (Table S2). Future research can investigate whether these two species have a facilitating influence on the root growth of nearby tree species. 

## 4. Materials and Methods

### 4.1. Site Description 

**Figure 2:**
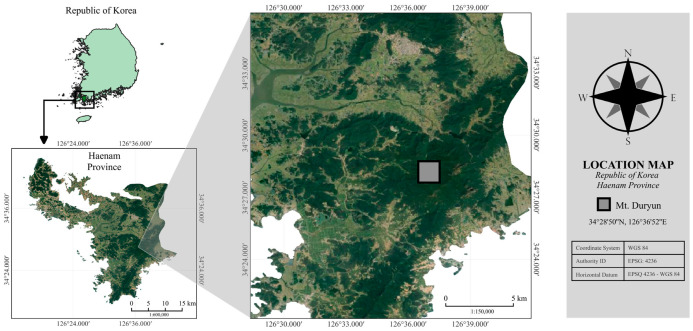
Geographical location of Mt. Duryun in the Haenam Province, Republic of Korea, where the experiment was conducted. All geospatial features are illustrated via EPSQ: 4236—WGS 84 horizontal datum.

This study was conducted in a warm-temperate forest located at Mt. Duryun in Haenam Province, Republic of Korea (34°28′50″ N, 126°36′52″ E) (Figure 2). Mt. Duryun, a 700 m high montane ecosystem on the south-western side of the Republic of Korea, forms diverse and well-defined communities of deciduous and evergreen forest trees. However, very limited research has been conducted in Mt. Duryun, particularly about the belowground system. 

With the unique assemblage of plant species distributed across different slope formations, Mt. Duryun is an ideal experimental site for investigating the extent and mechanisms underlying root biomass and distribution based on topography. The area is positioned at an altitude of 195 m and comprises a secondary successional forest that is home to a range of deciduous and evergreen broadleaf tree species [72]. The dominant climate type is humid subtropical (Cfa), with a long-term (1990–2022) normal temperature of 13.5 °C and total annual precipitation of 1273.5 mm. 

Two contrasting stands were designated as research sites based on topographic position, tree density, basal area (BA), tree diameter at breast height (DBH), and species diversity (Table 6). The first stand (hereafter, lower slope) is a relatively flat area oriented at ≤3°. The lower slope is distinguished by a distinct canopy stratum dominated by *Camellia japonica* L., *Cornus controversa* Hemsl., and *Ulmus davidiana* Planch. *C. japonica* occupies 44% of the total basal area (BA) at 11.9 m^2^ ha^−1^, followed by *C. controversa* and *U. davidiana* at 8.4 m^2^ ha^−1^ (24% of the total BA) and 6.7 m^2^ ha^−1^ (22% of the total BA), respectively. The second stand (hereafter, upper slope) is located 100 m north of the first stand and has a slope equivalent to 30°. The upper slope presents a clear vertical forest structure with a pronounced understory layer and a canopy layer populated by *Platycarya strobilacea* Siebold & Zucc., *Carpinus tschonoskii* Maxim., and *Camellia japonica* L. Unlike the lower slope, the upper slope has a relatively less variable BA distribution shared by *P. strobilacea* at 28% (14.6 m^2^ ha^−1^), *C. tschonoskii* at 16% (7.4 m^2^ ha^−1^), and *C. japonica* at 14% (7.2 m^2^ ha^−1^). 

### 4.2. Excavation of Soil Pits

Eleven 400 m^2^ sampling plots, five on the lower slope and six on the upper slope, about 20 m apart, were established at the research site. The distance between the research sites was maintained at 25 to 35 m to ensure that any observed results were independent of each other. A grid-like, wooden reference plate was randomly installed per plot, with no recent disturbances after the initial clearing of the organic layer. In each sampling plot, a 50 cm × 50 cm pit was excavated to a depth of 30 cm, with soil samples retrieved and separated into intervals of 0–10 cm, 10–20 cm, and 20–30 cm. Rocks were segregated from roots at each depth interval using a sieve with a 6 mm opening. The soil passing through the sieve was collected for further root processing. The cumulative volume of rock fragments (>6 mm) was measured directly via the water displacement method. 

### 4.3. Root Sorting and Biomass Measurement

The collected root samples were stored below freezing temperatures until further processing in the laboratory. Each sample was rinsed with tap water over a double-layered plastic net to minimize the loss of roots during washing. Thereafter, samples were air-dried and then subjected to visual inspection to eliminate dead roots. Dead fine roots were distinguished from their living counterparts by their brittle and dark exteriors [46]. The remaining root samples were stratified into four distinct sizes: 0–2 mm (fine), 2–5 mm (small), 5–10 mm (medium), and >10 mm (coarse) [53,73]. Root dry weight was measured for each diameter class using a standard laboratory balance after oven-drying at 65 °C for one week.

### 4.4. Effect of Size and Proximity of Nearby Trees 

The effect of proximity of nearby trees (stems with >2 cm diameter at breast height—DBH) on root biomass at each sampling site was determined using a tree influence index (Index_tree_) at various proximities and root diameter classes [46]. The tree influence index is denoted by the following equation:

$${\mathrm{Index}}_{\mathrm{tree}}=\sum_{i=1}^{t}\frac{{DBH}_{i}^{2}}{d_{i}}$$

where *t* is the number of trees within a specified distance from the pit, DBH*_i_* is the diameter of tree *i*, and d*_i_* is the distance of tree *i* from the excavation midpoint.

Trees within the first five-meter range of each pit were inventoried by recording their DBH and proximity to the excavation midpoint. Four proximities of 2 m, 3 m, 4 m, and 5 m were identified (Figure S1). Index_tree_ was determined for each range category by obtaining the sum of squares of all DBH estimates at a given range divided by the sum of their proximity to the excavation midpoint. The relationship between root biomass and Index_tree_ was analyzed for each sampling point. 

### 4.5. Soil Physicochemical Analysis 

The soil samples (N = 11) were collected from each research site for a soil physicochemical property analysis in the laboratory. The soil bulk density (BD) was determined for each sample using a core sampling method after drying the samples for three days at 105 °C. The total porosity was calculated using the equation 1-BD/particle density ×100 [74]. Soil texture (sand, silt, and clay) and organic matter (OM) were determined by the hydrometer method at 30 °C and the Tyurin method, respectively. Soil pH was estimated in a 1:5 (*w*/*v*) soil-distilled water suspension using a soil pH meter. Total nitrogen (TN) was extracted by digesting 1 g of soil into a concentrated H_2_SO_4_ solution using the micro-Kjeldahl method. Available phosphorus (P_2_O_5_; AP) was measured through the Lancaster method, and exchangeable cations (K^+^, Ca^2+^, and Mg^2+^) were analyzed using an atomic absorption spectrometer (AAS Varian, AA280FS, Santa Clara, CA, USA) after dissolving samples in a 1N NH_4_OAc extract. Cation exchange capacity (CEC) was analyzed in 1N NH_4_OAc and 1N CH_3_COOH solutions using the Brown method. Soil OM and nutrient content were calculated by multiplying nutrient concentration with bulk density and soil depth per research site.

### 4.6. Statistical Analyses 

The datasets were tested for normality and heterogeneity of variance using the Shapiro–Wilk test and Levene’s test, respectively. Soil physicochemical traits, fine root biomass, and rock distribution values were compared between different topographic positions and across depths using Quade’s non-parametric rank analysis of covariance, with species composition and stand structure treated as nuisance covariates. Independent pairwise comparisons were conducted for all significantly different means with more than two levels (i.e., depth) using Dunn’s Test. The resulting alpha values were adjusted using the Bonferroni correction to account for the number of performed pairwise comparisons and to reduce the likelihood of a Type 1 error. The same set of analyses was performed to identify differences in fine root biomass distribution by diameter class at each topographic position.

Linear-mixed effect models (LMMs) were generated to explore the relationships between fine root biomass and soil physicochemical traits for each diameter class. In this analysis, fine root biomass was considered as the response variable, soil physicochemical traits as fixed effect terms, and topography and species composition as random effect terms. The most parsimonious models were extracted using the dredge function of the MuMin package, and the standardized beta coefficient, standard error, and *p*-value of each fixed term were identified for each model.

A partial Spearman’s rank correlation was conducted to determine the strength of the relationship between fine root biomass and rock volume while adjusting for observation-level differences in species composition and stand structure. Rank correlation coefficients, along with their respective p-values, were determined for each diameter level and depth interval on the lower and upper slopes. The same approach was used to investigate the relationship between fine root biomass distribution and the surrounding trees at different levels of proximity. In this analysis, rank correlation coefficients were calculated at cumulative distances of 2, 3, 4, and 5 m, as well as within ranges of 2–3, 3–4, and 4–5 m. All analyses were performed in RStudio (version 4.2.3) at an α = 0.05 significance level.

## 5. Conclusions

The present study revealed interesting patterns in root biomass distribution influenced by topography, soil physicochemical properties, and tree influence. Here, topography-driven changes in soil physicochemical properties largely explain root biomass distribution along a gradient of soil depth. Moreover, both competitive and facilitative interactions also help elucidate the relationship between root biomass and the tree influence index across diameter classes and soil depths in Mt. Duryun in Republic of Korea. Therefore, our results have proven that the differences in the soil environment and topographical positions play crucial roles in shaping root biomass distribution across soil profile gradients. The findings of the present study will enhance our understanding of the different abiotic factors that determine belowground productivity. Future research can explore soil deeper layers (>30 cm) for a more comprehensive understanding of root–soil interactions and ecosystem functioning.

## Figures and Tables

**Table 1:** Soil physicochemical properties at the upper and lower slopes of warm-temperate forests in Mt. Duryun in Haenam Province, Republic of Korea.

Slope	Soil Depth	Variable	Physical Properties				Chemical Properties							
			BD	Sand	Silt	Clay	pH	OM	TN	AP	CEC	K^+^	Ca^2+^	Mg^2+^
			g cm^−3^	%	%	%		%	%	mg kg^−1^	clmo_c_ kg^−1^	clmo_c_ kg^−1^	clmo_c_ kg^−1^	clmo_c_ kg^−1^
Lower slope	0–10 cm	Median	0.81 B	12.21	71.89	14.29	5.84	8.31 A	0.55 A	19.46 A	22.05 A	0.57 A	8.38 A	2.69 A
		Mean	0.83 B	13.33	72.00	14.66	5.90	8.08 A	0.56 A	19.56 A	26.10 A	0.58 A	8.73 A	2.78 A
		SE	0.04	2.15	2.22	1.03	0.09	0.47	0.02	3.72	3.14	0.05	0.70	0.21
		Variance	0.01	28.06	29.60	6.34	0.04	1.29	0.01	83.48	59.43	0.01	2.92	0.26
	10–20 cm	Median	0.88 AB	11.55	72.32	17.02	5.76	6.63 AB	0.46 AB	9.54 AB	18.51 AB	0.36 AB	5.05 AB	1.87 AB
		Mean	0.86 AB	12.82	70.32	16.85	5.72	6.30 AB	0.45 AB	11.28 AB	17.65 AB	0.37 AB	5.30 AB	2.00 AB
		SE	0.09	2.45	2.66	1.06	0.07	0.41	0.02	1.24	1.38	0.03	0.60	0.23
		Variance	0.01	35.97	42.72	6.59	0.04	0.99	0.01	9.05	11.37	0.01	2.22	0.30
	20–30 cm	Median	1.03 A	11.04	70.35	17.83	5.78	4.83 B	0.35 B	9.49 B	15.26 B	0.28 B	3.54 B	1.50 B
		Mean	1.02 A	12.63	69.15	18.25	5.75	4.78 B	0.37 B	9.93 B	15.80 B	0.28 B	3.73 B	1.55 B
		SE	0.03	2.56	2.55	0.83	0.07	0.45	0.03	0.48	0.70	0.03	0.44	0.20
		Variance	0.01	39.21	38.91	4.14	0.03	1.21	0.01	1.35	2.86	0.01	1.13	0.22
Upper slope	0–10 cm	Median	0.73	7.92 B	74.67	17.99	5.73	6.01	0.48 A	10.53	20.77 A	0.47 A	5.99	2.62
		Mean	0.82	8.30 B	74.38	17.32	5.50	5.68	0.44 A	18.08	19.62 A	0.54 A	5.22	2.32
		SE	0.09	0.44	1.69	1.63	0.21	1.17	0.04	5.56	1.05	0.10	0.75	0.41
		Variance	0.04	0.90	14.22	13.16	0.25	6.87	0.01	155.01	5.48	0.05	2.75	0.79
	10–20 cm	Median	0.91	8.88 AB	69.82	20.56	5.55	5.45	0.30 AB	9.46	14.35 AB	0.29 AB	3.39	2.02
		Mean	0.93	9.30 AB	69.38	21.36	5.50	4.04	0.32 AB	9.96	14.96 AB	0.32 AB	3.02	1.64
		SE	0.06	0.34	1.31	1.10	0.24	0.79	0.04	0.46	0.68	0.05	0.71	0.40
		Variance	0.02	0.61	8.42	6.06	0.28	3.12	0.01	1.11	2.36	0.01	2.45	0.81
	20–30 cm	Median	1.12	10.26 A	70.00	20.56	5.58	2.79	0.20 B	9.42	12.92 B	0.25 B	1.85	1.45
		Mean	1.11	10.24 A	69.34	20.35	5.60	2.70	0.22 B	9.48	12.58 B	0.26 B	2.16	1.34
		SE	0.05	0.37	0.87	1.27	0.23	0.43	0.02	0.10	0.48	0.04	0.69	0.43
		Variance	0.01	0.64	3.78	4.88	0.28	0.91	0.01	0.05	1.18	0.01	2.33	0.89

BD, bulk density; OM, organic matter; TN, total nitrogen; AP, available phosphorus; CEC, cation exchange capacity; SE: standard error of the mean; different uppercase letters in a column indicate significant differences among soil depths within the same topographic position (*p* < 0.05).

**Table 2:** Linear mixed-effects models (LME) summarizing the influence of soil physicochemical properties on fine (0–2 mm), small (2–5 mm), medium (5–10 mm), and coarse roots (>10 mm) in the lower and upper slopes of warm-temperate forests of Mt. Duryun in Haenam Province, Republic of Korea.

Soil Physicochemical Traits	Root Diameter Class			
	Fine	Small	Medium	Coarse
*Physical properties*				
Bulk density	0.213 (0.002) **	0.702 (0.00002) ***	1.375 (0.167) *	−0.475 (0.001) ***
Sand		0.810 (0.00004) ***		1.216 (0.001) ***
Silt		0.070 (0.00003) ***		
Clay				
*Chemical properties*				
pH	−1.036 (0.003) **	−0.130 (0.00003) ***	−0.938 (0.192)	3.732 (0.001) ***
OM	0.864 (0.003) **	2.440 (0.00003) ***	3.203 (0.346) *	−2.752 (0.003) ***
TN	−1.335 (0.005) **		−6.302 (0.527) *	−0.437 (0.002) **
AP	0.136 (0.002) **	−1.123 (0.00002) ***	1.012 (0.151)	
CEC	−1.005 (0.001) ***	−1.517 (0.00002) ***	−0.436 (0.120)	0.496 (0.001) ***
K^+^	0.837 (0.004) **		3.003 (0.347)	3.038 (0.001) ***
Ca^2+^				
Mg^2+^				

OM, organic matter; TN, total nitrogen; AP, available phosphorus; CEC, cation exchange capacity. Numeric values indicate standardized beta coefficients; values enclosed in parenthesis represent standard error of coefficients; *, **, and *** denote statistical significance at *p* < 0.05, *p* < 0.01, and *p* < 0.001, respectively.

**Table 3:** Depthwise distribution of root biomass (g m^2^) across diameter classes on the upper and lower slopes of Mt. Duryun in Haenam Province, Republic of Korea.

Root Diameter Class	Variable	Lower Slope			Upper Slope		
		0–10 cm	10–20 cm	20–30 cm	0–10 cm	10–20 cm	20–30 cm
Fine root	Median	38.84	41.44	24.66	65.64	52.00	39.84
	Mean	38.67	42.21	24.93	57.80	43.86	31.52
	Variance	385.92	299.07	75.78	320.61	228.64	301.85
	SE	8.02	7.05	3.56	8.00	6.75	7.77
Small root	Median	19.24 b	50.10 a	39.78 ab	52.56	46.08	29.84
	Mean	19.65 b	49.34 a	46.75 ab	52.62	54.78	38.78
	Variance	169.40	239.15	375.85	670.00	482.03	457.08
	SE	5.31	6.30	7.90	11.57	9.82	9.56
Medium root	Median	7.48	42.64	40.44	56.04	69.52	32.56
	Mean	14.33	39.21	40.11	50.32	62.98	36.74
	Variance	342.75	542.12	804.83	502.51	1261.15	440.68
	SE	7.55	9.50	11.59	10.02	15.87	9.38
Coarse root	Median	0.00 b	66.44 ab	141.08 a	52.84	121.04	75.52
	Mean	12.96 b	97.50 ab	187.65 a	43.47	150.90	257.95
	Variance	1007.77	12,668.97	35,725.21	1719.11	17,705.20	93,240.37
	SE	12.96	45.94	77.15	18.54	59.49	136.55

SE, standard error of the mean; different lowercase letters in a row indicate significant differences among soil depths within the same topographic position (*p* < 0.05).

**Table 4:** Partial Spearman rank correlation coefficients describing the strength of fine (0–2 mm), small (2–5 mm), medium (5–10 mm), and coarse root (>10 mm) biomass relationships with rock distribution in warm-temperate forests in the lower and upper slopes of Mt. Duryun in Haenam Province, Republic of Korea.

Slope/Soil Depth (cm)	Root Diameter Class			
	Fine	Small	Medium	Coarse
*Lower Slope*				
0–10	−0.34	−0.53	−0.72	−0.72
10–20	0.34	−0.06	0.00	0.41
20–30	0.14	−0.07	−0.48	0.00
*Upper slope*				
0–10	−0.70	−0.10	0.24	0.19
10–20	−0.90	−0.84	−0.84	0.58
20–30	−0.82	−0.58	0.40	0.67

**Table 5:** Partial Spearman rank correlation coefficients describing the strength of fine (0–2 mm), small (2–5 mm), medium (5–10 mm), and coarse root (>10 mm) biomass correlation with tree influence index at different proximity from the excavation midpoint in warm-temperate forests in the lower and upper slopes of Mt. Duryun in Haenam Province, Republic of Korea.

Slope/Diameter Class	Distance (m)						
	2	3	4	5	2–3	3–4	4–5
*Lower slope*							
Fine	−0.24	−0.10	−0.09	−0.14	−0.27	−0.06	−0.05
Small	−0.01	−0.06	−0.16	0.17	−0.02	−0.10	−0.14
Medium	−0.27	−0.15	−0.46	0.34	−0.30	−0.34	−0.46
Coarse	−0.06	0.25	0.04	−0.14	0.07	0.26	−0.06
*Upper slope*							
Fine	0.68 **	0.25	−0.50	−0.72 ***	0.71 ***	−0.08	−0.78 ***
Small	−0.03	0.84 ***	0.14	−0.06	0.11	0.59 *	−0.35
Medium	0.15	0.26	−0.13	−0.18	0.17	0.07	−0.26
Coarse	−0.45	0.47	0.33	0.41	−0.39	0.39	0.24

*, **, and *** denote statistical significance at *p* < 0.05, *p* < 0.01, and *p* < 0.001, respectively.

**Table 6:** Stand structure of warm-temperate forests in the lower and upper slopes of Mt. Duryun in Haenam Province, Republic of Korea.

Topographic Position/Slope		Lower	Upper
Tree density (tree ha^−1^)	Mean	1125	3847
	Variance	197,438	765,953
	SE	180	388
Basal area (m^2^ ha^−1^)	Mean	30.1	49.9
	Variance	88.0	142.9
	SE	3.8	5.3
Diameter at breast height (cm)	Mean	16.3	8.8
	Variance	18.2	3.6
	SE	1.7	0.8
Shannon–Wiener index	Mean	0.8	1.8
	Variance	0.1	0.3
	SE	0.1	0.2

SE: standard error of the mean.

## Data Availability

The data presented in this study are available within the article.

## Supplementary Materials

The following supporting information can be downloaded at: https://www.mdpi.com/article/10.3390/plants13101340/s1, Table S1. Volumetric rock fragment content (%) on the lower and upper slopes of Mt. Duryun in Haenam Province, Republic of Korea; Table S2. Mean Diameter at Breast Height (DBH) of dominant tree species in relation to various distances from the excavated pit in the lower and upper slopes in Mt. Duryun, Haenam Province, Republic of Korea; Figure S1. Distances from midpoints (pit) excavated in the research site in Mt. Duryun, Haenam Province, Republic of Korea.
