# Supplementary material for: Variation in Root Biomass and Distribution Based on the Topography, Soil Properties, and Tree Influence Index: The Case of Mt. Duryun in Republic of Korea
Source: Plants (Basel). 2024 May 13;13(10):1340. doi: 10.3390/plants13101340 (PMC11125356; doi:10.3390/plants13101340)
Supplement: Supplementary file 1 [file plants-13-01340-s001.zip › plants-2985316-supplementary.pdf]

Supplementary Information

Table S1. Volumetric rock fragment content (%) on the lower and upper slopes of Mt. Duryun in Haenam Province, Republic of Korea

Soil depth (cm)	Lower slope	Upper slope
0-10	27.90	29.32
10-20	19.40	28.60
20-30	27.26	20.40

The central tendency of rock distribution is expressed through the median (n = 5–6)

Table S2. Mean Diameter at Breast Height (DBH) of dominant tree species in relation to various distances from the excavated pit in the lower and upper slopes in Mt. Duryun, Haenam Province, Republic of Korea

Slope	Distance from the pit (m)	Dominant species	Mean DBH of dominant species (cm)
Lower	2	*Camellia japonica L.*<br>*Cornus controversa Hemsl.*<br>*Hovenia dulcis Thunb.*	12.73<br>24.00<br>13.50
	3	*Camellia japonica L.*<br>*Cornus controversa Hemsl*<br>*Hovenia dulcis Thunb.*	14.07<br>25.80<br>13.50
	4	*Camellia japonica L.*<br>*Cornus controversa Hemsl.*<br>*Hovenia dulcis Thunb.*<br>*Zelkova serrata (Thunb.) Makino*	10.13<br>23.09<br>13.50<br>30.10
	5	*Camellia japonica L.*<br>*Cornus controversa Hemsl.*<br>*Zelkova serrata (Thunb.) Makino*<br>*Ulmus davidiana Planch. var. Japonica*<br>*Hovenia dulcis Thunb.*	10.84<br>23.09<br>28.85<br>44.70<br>13.50
	2-3	*Camellia japonica L.*<br>*Cornus controversa Hemsl*	14.23<br>27.60
	3-4	*Camellia japonica L.*<br>*Cornus controversa Hemsl*<br>*Zelkova serrata (Thunb.) Makino*	7.03<br>20.38<br>30.10
	4-5	*Camellia japonica L.*	11.34

		Ulmus davidiana Planch. var. Japonica	44.70
		Zelkova serrata (Thunb.) Makino	27.60
Upper	2	*Camellia japonica L.*	9.32
		Cinnamomum japonicum Siebold	5.83
		Platycarya strobilacea Siebold & Zucc.	28.65
		Carpinus laxiflora (Siebold & Zucc.) Blume	5.00
		Quercus salicina Blume	34.40
	3	*Camellia japonica L.*	7.71
		Cinnamomum japonicum Siebold	5.66
		Carpinus laxiflora (Siebold & Zucc.) Blume	10.83
		Neoshirakia japonica (Siebold & Zucc.) Esser	3.90
		Platycarya strobilacea Siebold & Zucc.	28.65
	4	*Camellia japonica L.*	6.90
		Cinnamomum japonicum Siebold	5.66
		Carpinus laxiflora (Siebold & Zucc.) Blume	10.83
		Carpinus tschonoskii Maxim.	15.90
		Neoshirakia japonica (Siebold & Zucc.) Esser	3.83
	5	*Camellia japonica L.*	6.28
		Cinnamomum japonicum Siebold	5.41
		Carpinus tschonoskii Maxim.	13.56
		Ficus erecta Thunb.	4.31
		Platycarya strobilacea Siebold & Zucc.	24.00
	2-3	*Camellia japonica L.*	6.74
		Carpinus laxiflora (Siebold & Zucc.) Blume	12.77
		Neoshirakia japonica (Siebold & Zucc.) Esser	3.90
		Eurya japonica Thunb.	7.85
		Symplocos tanakana Nakai	5.90
	3-4	*Camellia japonica L.*	6.02
		Cinnamomum japonicum Siebold	5.66
		Carpinus tschonoskii Maxim.	15.67
		Toxicodendron sylvestre (Siebold & Zucc.) Kuntze	11.30
		Prunus jamasakura Siebold ex Koidz.	10.45
	4-5	*Camellia japonica L.*	5.87
		Cinnamomum japonicum Siebold	5.20
		Carpinus laxiflora (Siebold & Zucc.) Blume	11.44
		Ficus erecta Thunb.	4.94
		Carpinus tschonoskii Maxim.	12.62

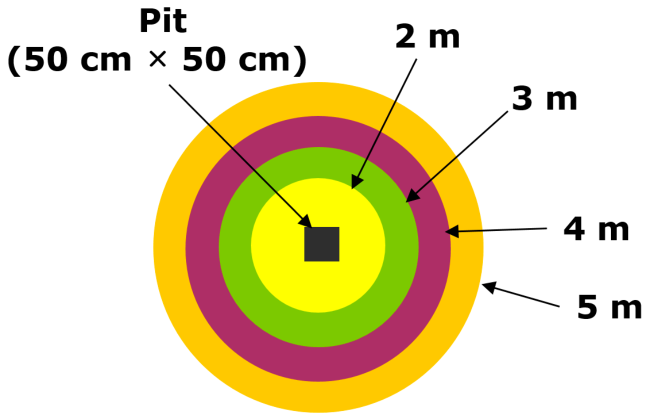

Figure S1. Distances from midpoints (pit) excavated in the research site in Mt. Duryun, Haenam Province, Republic of Korea
